# Polylactic acid composites incorporating casein functionalized cellulose nanowhiskers

**DOI:** 10.1186/1754-1611-7-31

**Published:** 2013-12-16

**Authors:** Jin Gu, Jeffrey M Catchmark

**Affiliations:** 1 Intercollege Graduate Degree Program in Plant Biology, The Pennsylvania State University, University Park, PA 16802, USA; 2 Department of Agricultural and Biological Engineering, The Pennsylvania State University, 109 Ag. Eng. Bldg., University Park, PA 16802, USA

**Keywords:** Cellulose nanowhiskers, Whole milk casein, Polylactic acid, Surface plasmon resonance, Fluorescence microscopy

## Abstract

**Background:**

Polylactic acid (PLA) is considered to be a sustainable alternative to petroleum-based polymers for many applications. Using cellulose fiber to reinforce PLA is of great interest recently due to its complete biodegradability and potential improvement of the mechanical performance. However, the dispersion of hydrophilic cellulose fibers in the hydrophobic polymer matrix is usually poor without using hazardous surfactants. The goal of this study was to develop homogenously dispersed cellulose nanowhisker (CNW) reinforced PLA composites using whole milk casein protein, which is an environmentally compatible dispersant.

**Results:**

In this study, whole milk casein was chosen as a dispersant in the PLA-CNW system because of its potential to interact with the PLA matrix and cellulose. The affinity of casein to PLA was studied by surface plasmon resonance (SPR) imaging. CNWs were functionalized with casein and used as reinforcements to make PLA composites. Fluorescent staining of CNWs in the PLA matrix was implemented as a novel and simple way to analyze the dispersion of the reinforcements. The dispersion of CNWs in PLA was improved when casein was present. The mechanical properties of the composites were studied experimentally. Compared to pure PLA, the PLA composites had higher Young’s modulus. Casein (CS) functionalized CNW reinforced PLA (PLA-CS-CNW) at 2 wt% filler content maintained higher strain at break compared to normal CNW reinforced PLA (PLA-CNW). The Young’s modulus of PLA-CS-CNW composites was also higher than that of PLA-CNW composites at higher filler content. However, all composites exhibited lower strain at break and tensile strength at high filler content.

**Conclusions:**

The presence of whole milk casein improved the dispersion of CNWs in the PLA matrix. The improved dispersion of CNWs provided higher modulus of the PLA composites at higher reinforcement loading and maintained the strain and stress at break of the composites at relatively low reinforcement loading. The affinity of the dispersant to PLA is important for the ultimate strength and stiffness of the composites.

## Introduction

The development of biodegradable polymer composites, especially composites combining biodegradable matrices and structural biodegradable reinforcements, is of great importance currently. Among these polymer matrices, polylactic acid (PLA) is being used widely in automobile, food packaging and pharmaceutical industries due to its high mechanical performance [1]. It is a commercially available polyester polymerized from lactic acid, which is produced from biorenewable sugar based sources, such as corn starch and sugarcane.

Nature fiber such as lignocellulose or purified cellulose derived from a variety of nature feedstocks (e.g. woods, cotton, and bacterial cellulose) has great potential to replace synthetic reinforcements such as glass fiber or fiber produced using petroleum derived polymers used in composite materials. To this end, various forms of cellulose including microcrystalline cellulose (MCC) or nanodimensional cellulose such as cellulose nanowhiskers (CNWs) and cellulose nanofibrils (CNFs) have been explored as reinforcements for different composite materials [2]. These celluloses are typically derived from controlled acid hydrolysis or mechanical homogenization of plant materials [3]. During the acid hydrolysis, the amorphous regions of the CNWs were preferentially removed. They are of particular interest because of their desirable mechanical properties (Young’s modulus of crystal cellulose is in the range of 100–160 GPa [4]) and bio-availability.

Natural fiber additives are challenging to incorporate into non-polar matrix materials due to their polar surface chemistries. This situation results in poor dispersion of the fiber in the matrix and poor interfacial adhesion limiting improvements in mechanical properties. This has been found to be true for PLA-nanocellulose composites [1,5].

To address these issues, different processing techniques have been implemented including solvent casting [5], premixing the materials before processing [1,6], addition of surfactant/plasticizer to improve the dispersion [7-10] and chemical modification of the nanofibers [11,12]. Generally speaking, solvent casting of cellulose nanocomposites results in more homogeneous materials than other industrially used processing techniques such as melt compounding [2]. However, the above processing techniques have used hazardous chemicals or could not achieve good dispersion. Such approaches would retract significantly from the ecological benefits of using PLA/cellulose composites.

Low cost agricultural products such as proteins from soy bean, corn and milk have been used to make plastic since the first half of the last century [13]. The milk proteins, named caseins, consist of four protein subunits (α_s1_, α_s2_, β and κ). Caseins are amphiphilic containing both hydrophobic and hydrophilic domains. They are distinct proteins which are able to disperse, solubilize and compatibilize the insoluble components (colloidal calcium phosphate, CCP) into an incompatible solution (water) by a soluble micelle [14-16]. Casein was extensively used as a wood adhesive before 1930’s. Its affinity to cellulose has been proposed to be associated with the interaction between the hydroxyl groups of cellulose and the peptide bonds of casein [17,18]. The proposed binding mechanism also involves carbohydrate-π interactions [19,20].

Inspired by the unique amphiphilic nature of casein proteins, this work aimed to explore the possibility of using ecologically compatible caseins as dispersants of cellulose into PLA. Surface plasmon resonance (SPR) imaging was used to characterize the interaction of PLA and the whole milk casein. The affinity of PLA to casein coated surfaces was studied in order to demonstrate the functionality of casein as a dispersant in the PLA matrix. Subsequently, casein functionalized CNWs-PLA composites were prepared using solvent casting method. The dispersion of CNWs in PLA was studied by staining the CNWs with Calcofluor White, which is a fluorescence dye known to bind cellulose, in a simple and effective manner compared to previous characterization methods such as electron microscopy [8]. The effect of fillers on the mechanical properties and crystallinity of the composites was evaluated by tensile testing and X-ray diffraction, respectively.

## Results and discussion

### Affinity of PLA to whole milk casein

**Figure 1:**
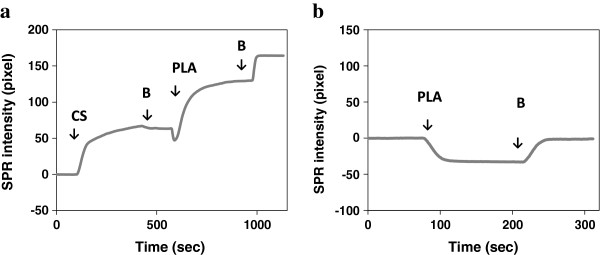
**SPR results at a fixed angle. (a)** The change of SPR intensity when the casein solution was flowed across the gold surface, and followed by 1 mM PLA solution. **(b)** Control experiment showing little change of final SPR intensity when PLA was flowed across the gold surface. The gold film was first exposed to buffer **(B)**, then casein (CS) or PLA solution. Arrows indicate the times when the solutions were changed.

Figure 1a depicts a typical SPR result for casein adsorption onto gold surfaces and PLA adsorption onto casein coated surfaces. Arrows on the figure indicate the times when the solutions were changed. This result is repeatable [21]. When the casein solution was flowed across the surface, an increase in SPR intensity was observed due to the adsorption of casein molecules onto the gold surface. Flow of SPR buffer on the casein coated surface caused a decrease of SPR intensity which indicated some reversibly bound casein left the surface. The coverage of casein reached equilibrium after the surface was exposed to the running buffer for about 1 minute.

**Figure 2:**
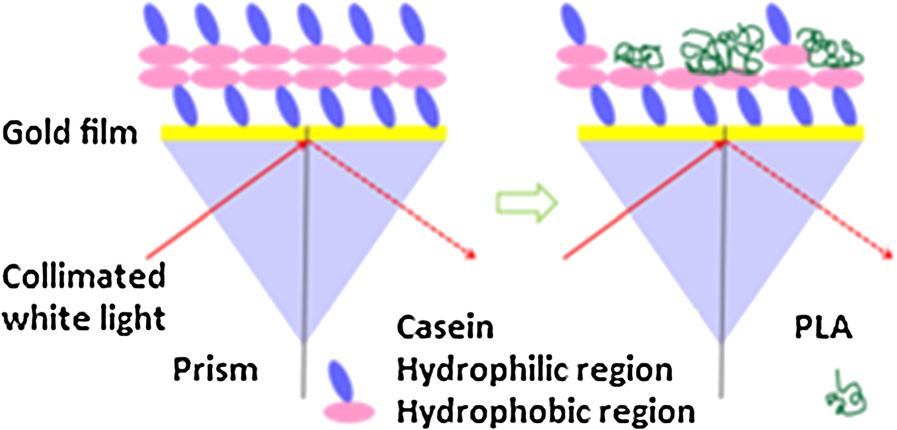
Schematic illustration of the SPR experiment showing deposition of casein onto the gold surface (left) and adsorption of PLA onto the casein coated surface (right).

The mechanism of casein adsorption onto gold surfaces has been studied previously. Liu et al. [22] proposed that the carboxylate or amine groups of the caseins exhibited an affinity to gold particles. In previous works [23-25], it was demonstrated that casein was able to form a bilayer on hydrophilic surfaces. Caseins behave like copolymers consisting of blocks containing hydrophobic and hydrophilic domains. The hydrophilic domains of caseins interacted with the hydrophilic gold surface causing the hydrophobic domains to orient away from the surface. The hydrophobic domains were then exposed to the solution allowing the adsorption of a second layer of casein through hydrophobic interactions (Figure 2). Using quartz crystal microbalance measurements, Ozeki et al. [24] stated that the first layer of casein on the hydrophilic surface was tightly bound and the second layer was reversibly bound. The decrease of SPR intensity when SPR buffer was flowed across the casein coated surface could be explained by desorption of the reversibly bound casein layer.

To study the interaction between immobilized casein and PLA, a PLA solution was flowed across the casein coated surface. As indicated in Figure 1a, the SPR intensity was increased by 70 units (arbitrary intensity units) in about two hundreds seconds. The initial decrease of SPR intensity was due to a change in refractive index of the PLA solution. After adsorption of PLA to the casein, the SPR intensity began to increase. The curve became flat after a steep increase indicating saturation of the binding. The adsorption of PLA to casein reached equilibrium after about three hundred seconds. Finally, the SPR buffer was again flowed across the surface. The increase of SPR intensity further confirmed that the refractive index of the buffer was higher than that of the PLA solution. To demonstrate that PLA did not interact with the gold surface and the overall increase of SPR intensity in Figure 1a was caused by casein-PLA interaction, a control experiment was performed. A PLA solution was flowed across the gold surface without a casein coating. Almost no net change of SPR intensity was observed when SPR buffer was flowed after exposure of the PLA solution to the gold surface (Figure 1b). The change of intensity from PLA to buffer in Figure 1b was also comparable to that in Figure 1a.

PLA is a non-polar and hydrophobic polymer. A hydrophobic interaction between PLA and casein occurred when PLA solution was flowed across this surface. Figure 2 shows a schematic illustration of the SPR imaging experiments. Casein was able to bind to gold through its hydrophilic domains. A reversibly bound casein layer was deposited on the first casein layer through hydrophobic interactions. After flowing in the casein-free buffer, some casein in the second layer left the surface and the hydrophobic domain of the casein was exposed. PLA was then able to interact with the hydrophobic domain of casein.

**Figure 3:**
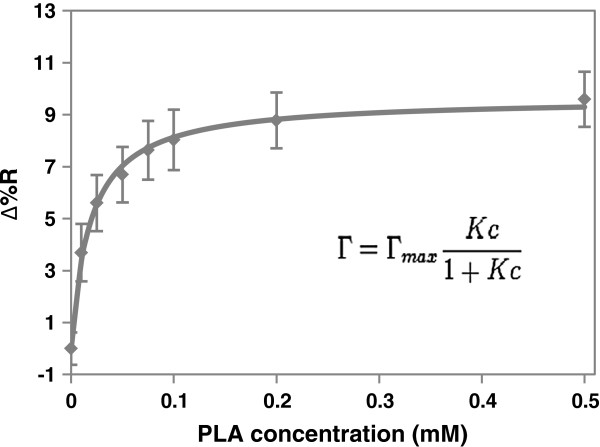
**Change in relative reflectivity value when 0.01, 0.025, 0.05, 0.075, 0.1, 0.2, 0.25, 0.5 mM PLA solutions were flowed across the casein coated surface in sequence.** Data is presented as mean standard deviation of five determinations and fit to the Langmuir isotherm (solid line).

To study the strength of interaction between casein and PLA, PLA solutions with different concentrations (from 0.01-0.5 mM) were flowed across casein coated surfaces in sequence. The SPR intensity was recorded at equilibrium and converted to a change in relative reflectivity value as shown in Figure 3 (See Equation 1 in Materials and methods). The trend reached a saturation point at 0.5 mM PLA. The data was fitted to a Langmuir isotherm. The adsorption coefficient and the maximum change of relative reflectivity were calculated by Equation 2. The adsorption coefficient for PLA binding to a casein coated surface was determined to be 5.40 ± 0.51 × 10^4^ M^-1^. The maximum change of relative reflectivity value was 9.64 ± 0.20%. The R^2^ of the fitting was as high as 0.98 which indicated Langmuir isotherm could best describe the adsorption behavior. This also suggested that PLA only formed a monolayer on the casein coated surface. The adsorption coefficient indicated a weak interaction between PLA and casein (compared to other protein carbohydrate interaction [26], where K is around 10^6^-10^7^ M^-1^).

### Distribution of fillers in composites

**Figure 4:**
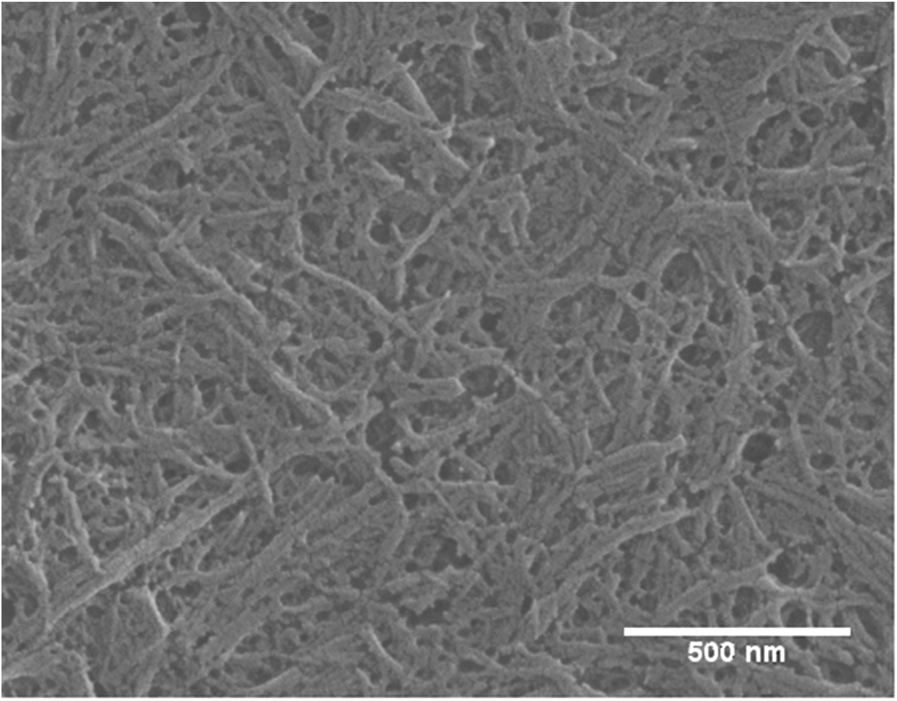
SEM image of CNWs prepared with sulfuric acid.

CNWs were highly crystalline nanofibers obtained by the acid hydrolysis of cotton cellulose. Stable colloidal suspensions were obtained because sulfuric acid treated cellulose carried negative charged sulfate groups [3]. Most CNWs were found to have lengths of approximately 200 nm to 300 nm with diameters of 15–30 nm (Figure 4, also determined previously [27]). Larger particles measuring several micrometers long which were not completely hydrolyzed were also observed in small amounts. The crystallinity of CNWs was as high as approximately 90% as studied previously [28]. Both freeze-dried CNWs and casein (CS) functionalized CNWs (CS-CNWs) were able to suspend in dimethylformamide (DMF) solution. DMF was selected as the solvent in this study due to its low toxicity (compared to solvents used in other studies, such as chloroform), ability to suspend CNWs, and dissolve PLA. Samir et al. [29] pointed out that the high value of the dielectric constant of DMF and its wettability on CNWs resulted in stable CNW suspensions. This method increased the dispersion of CNWs in the PLA matrix as compared to other solvents such as acetone or chloroform.

**Figure 5:**
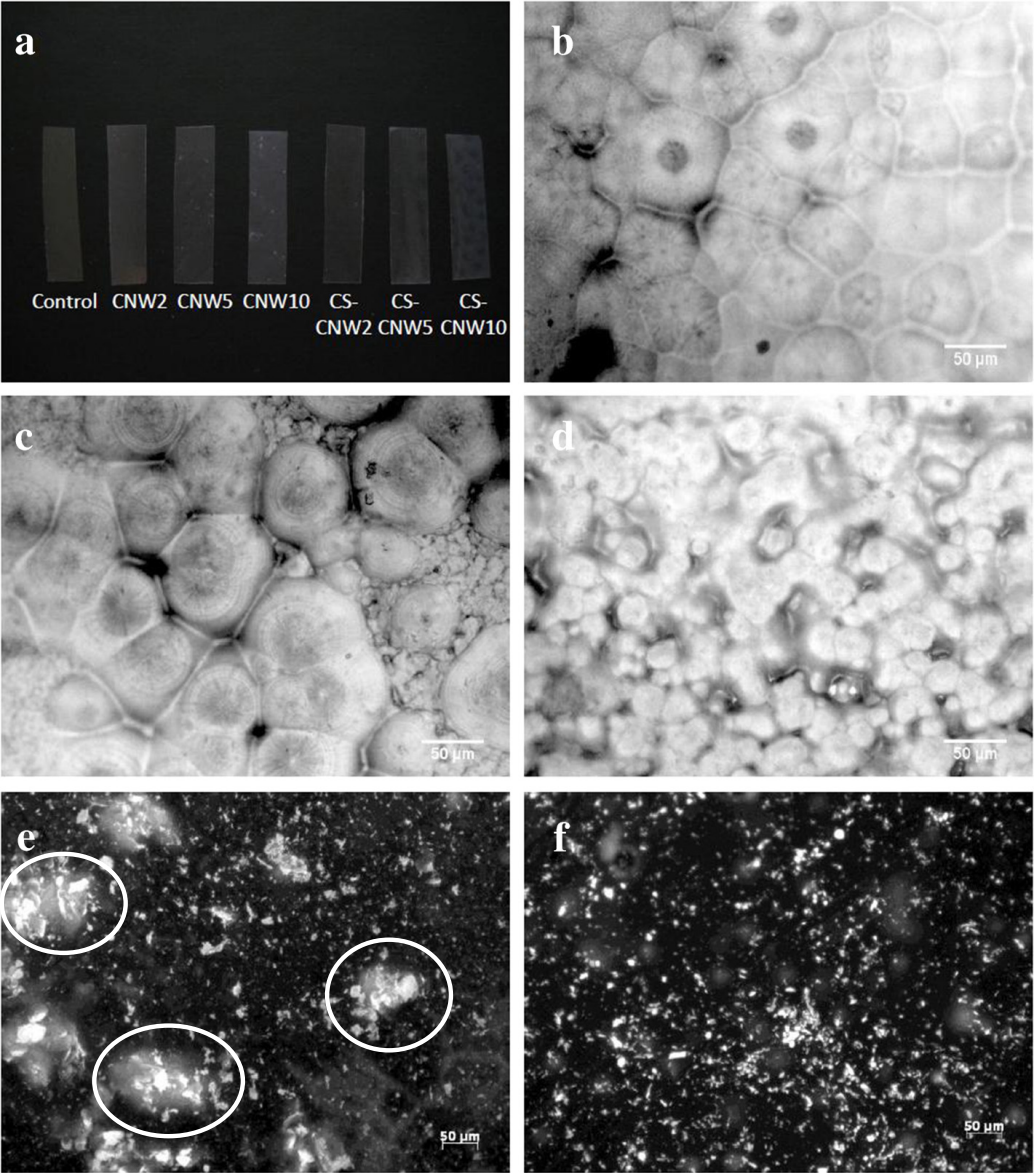
**Visual comparison of PLA and its composites. (a)** Image showing the appearance of different samples. **(b-d)** Optical micrographs of the materials: **(b)** PLA; **(c)** PLA-CNW2; and **(d)** PLA-CS-CNW2. **(e-f)** Fluorescent images of the composites: **(e)** PLA-CNW2; **(f)** PLA-CS-CNW2. The aggregations of CNWs are circled in **(e)**.

Photographs of PLA films with different reinforcement concentrations are presented in Figure 5a. The pure PLA control and PLA with 2% CNW (CNW2) or with 2% CS-CNW (CS-CNW2) were transparent. PLA films with 5% or 10% CNW (CNW5 and CNW10) exhibited some white spots which may be the CNWs aggregates. The transparency of the PLA films containing CS-CNW was decreased with 5% CS-CNW (CS-CNW5), and even more with 10% CS-CNW(CS-CNW10) filler content, but few white spots were observed.

In order to further understand the distributions of fillers in the PLA composites, the optical microscope images of PLA, PLA-CNW2 and PLA-CS-CNW2 were obtained as shown in Figure 5b,c,d. When the DMF solvent evaporated, single PLA crystals were formed which subsequently arranged themselves into large, sphere-like structures, called spherulites. Spherulites exhibiting diameters of approximately 50 microns formed in pure PLA films. In PLA-CNW2, large spherulites with similar size to those observed in pure PLA films were found together with many additional smaller spherulites. Medium size spherulites with 20–30 μm diameters were observed in PLA-CS-CNW2 composites. Since nucleation of PLA on the reinforcement surface was observed previously in composites [30], the change of spherulite size in the PLA-CS-CNW2 composite could possibly result from the presence of the fillers. The tiny spherulites in PLA-CNW2 may grow on the surface of CNW aggregates. In PLA-CS-CNW2, since PLA had higher affinity to the casein-coated fillers, the CS-CNWs may be better nucleating agents. Also, the distribution of filler was more uniform in the PLA-CS-CNW2, resulting in a larger number of smaller spherulites.

To further understand the distribution of the filler, the composite films with Calcofluor staining (see Materials and methods section) were investigated as shown in Figure 5e, f. Agglomerates of CNWs were discernible by their higher local fluorescence intensity. PLA-CNW2 composite showed randomly distributed bright aggregation spots. The aggregations of CNWs are highlighted with circles in Figure 5e. PLA-CS-CNW2 composite, however, showed evenly distribution of spots (Figure 5f). It suggests that the CS-CNWs were better dispersed in the PLA matrix.

The sodium hydroxide in the stain dissolved the PLA polymer allowing the surface of CNWs to adsorb Calcofluor and became fluorescent. In alkaline condition, the crystallinity of the PLA was first increased and then decreased [31]. The hydrolytic degradation was a two-stage process. It indicated that the amorphous part of the PLA was hydrolyzed first. During our short experimental time, no significant erosion of the composite was observed. This is a simple method to observe the dispersion of CNWs in PLA matrix compared to other methods, such as electron microscopy and atomic force microscopy [8]. Prior work successfully developed a method for fluorescent labeling of CNWs [32], but the chemical modification of CNWs may have an impact on the filler-matrix or filler-filler interaction.

### Mechanical properties of PLA composites

**Figure 6:**
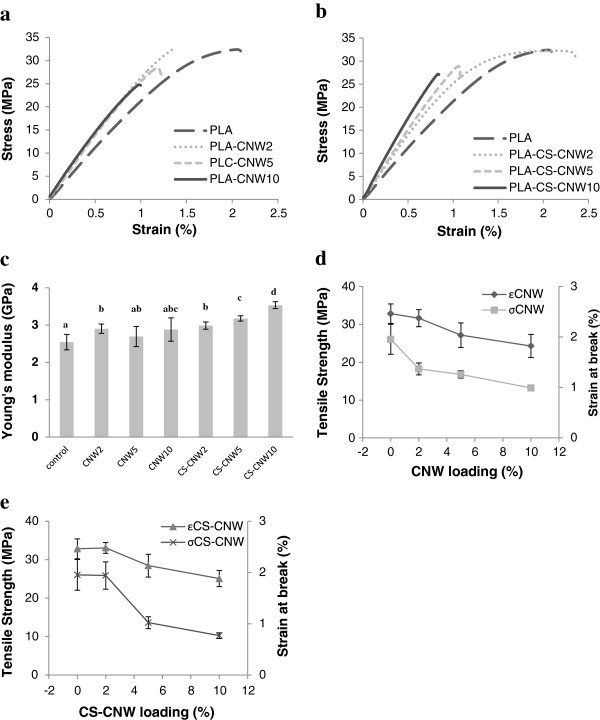
**Mechanical properties of PLA and its composites. (a-b)** Typical strain–stress curves of PLA and PLA composites: **(a)** PLA-CNW composites and **(b)** PLA-CS-CNW composites; **(c)** Young’s modulus of all samples. T-test results are displayed as letters on the top of columns. A column with the same letter represents no significant difference (p > 0.05). **(d)** Tensile strength and strain at break of PLA-CNW composites; **(e)** Tensile strength and strain at break of PLA-CS-CNW composites. ϵ represents stress. σ represents strain.

Figure 6a,b shows typical tensile curves for pure PLA and PLA composites. Data extracted from these plots are presented in Figure 6c,d,e and Table 1 with mean and standard deviations. Values of the Young’s modulus which are different based on statistical analysis (p < 0.05) are identified (small letters) in Figure 6c. It is clearly showed that the addition of CNWs had a positive effect on the Young’s modulus of the composites. The effect of reinforcement was achieved even with only 2 wt% CNWs. The Young’s modulus of PLA composites was increased from 2.5 GPa to roughly 3.0 GPa at 2 wt% filler loading. Compared to PLA-CNW composites, PLA-CS-CNW composites showed higher Young’s modulus with increasing filler contents. The Young’s modulus was 3.5 GPa for PLA-CS-CNW10 compared to 2.9 GPa for PLA-CNW10. The standard deviations were higher for PLA-CNW composites which may result from the inhomogeneous distribution of the CNW filler in the matrix. As shown in Figure 5, the distribution of fillers was better in PLA-CS-CNW composites, which may result in improved reinforcement. Moreover, the improved modulus may also result from changes in spherulite formation. The CS-CNW composite exhibited more numerous, smaller spherulites.

**Table 1:** Mechanical properties and crystallinity of PLA and PLA composites

**Samples**	**Young’s modulus (GPa)**	**Tensile strength (MPa)**	**Strain at break (%)**	**Crystallinity (%) (XRD)**
PLA	2.5 ± 0.2	32.8 ± 2.6	2.0 ± 0.3	47.8 ± 4.1
PLA-CNW2	2.9 ± 0.1	31.6 ± 2.3	1.4 ± 0.1	48.6 ± 0.5
PLA-CNW5	2.7 ± 0.3	27.1 ± 3.2	1.3 ± 0.1	43.2 ± 0.7
PLA-CNW10	2.9 ± 0.3	24.3 ± 3.0	1.0 ± 0.1	37.1 ± 1.8
PLA-CS-CNW2	3.0 ± 0.1	33.1 ± 1.4	1.9 ± 0.3	41.7 ± 1.3
PLA-CS-CNW5	3.2 ± 0.1	28.4 ± 3.0	1.0 ± 0.1	44.8 ± 4.4
PLA-CS-CNW10	3.5 ± 0.1	25.1 ± 2.1	0.8 ± 0.1	40.1 ± 1.3

The strain at break of the composites was generally lower than that of pure PLA with one exception which was PLA-CS-CNW2. It is a common observation that the addition of fibers to the polymer reduces the elongation at break in thermoplastic composites [1,33]. At low CS-CNW content, the better adhesion of CS-CNW to the matrix may reduce the voids at the fiber matrix interface and thus the matrix could elongate as pure PLA. Also, with decreased spherulite size in PLA-CS-CNW2, the strain at failure, tensile strength and toughness could increase as shown in other polymer [34]. At higher CS-CNW content, the strain at break decreased. The decrease was more significant when compared to the PLA-CNW composites, suggesting that the casein was effective at improving the bonding of the CNWs to PLA. Since the filler only contained 0.1% casein (data shown in Materials and methods), the CNW surface may not be fully covered by the casein, possibly suggesting that improved coating of the casein on the CNWs or other fillers may further improve performance. As the distribution of CS-CNW was better than CNW, more interface area between filler and matrix may be expected and thus the fracture initiating points may increase and strongly influence the mechanical properties of the composites. Another possibility is that a small amount of free casein protein was present in the matrix causing brittleness of the composites, since protein itself can be brittle [35]. This is unlikely in the current study, however, since the total amount of casein in the composite did not exceed 0.01% w/w (CS:PLA) in the case of highest CNW loading (10%).

The tensile strength was comparable for pure PLA and PLA composites with 2% filler loading. At higher filler loading, the tensile strength was decreased for both fillers types. It decreased even more for PLA-CNW composites. The decrease of strength in the composites is likely due to poor stress transfer across the interface [33].

### Effect of fillers on the crystallinity of polylactic acid

**Figure 7:**
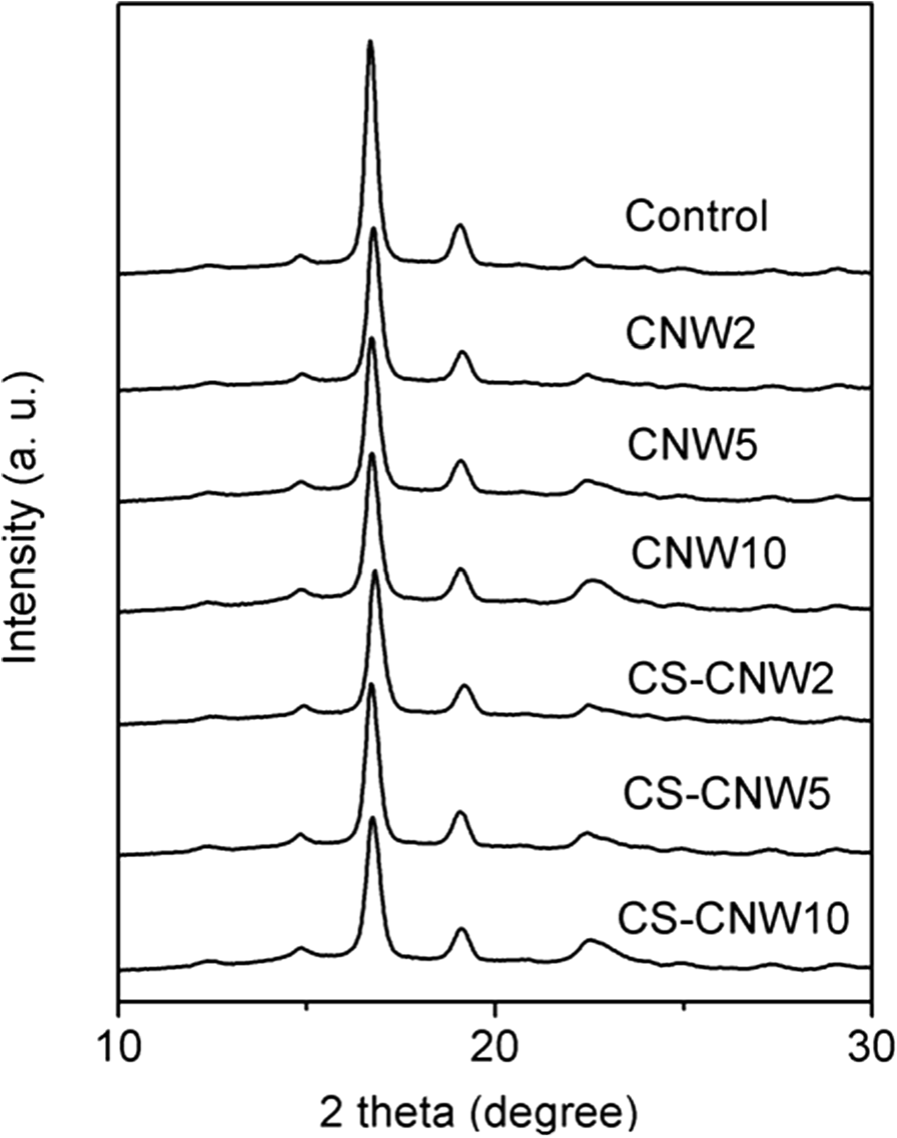
X-ray diffraction patterns of PLA and its composites.

Figure 7 shows the X-ray diffraction pattern of PLA and PLA composites. Four crystalline PLA peaks at 14.8, 16.4, 19.0 and 22.3° 2θ are obvious. A broad peak at around 17° 2θ showed the amorphous portion of the PLA. In PLA composites, a cellulose peak at around 22.7° 2θ could be found and its intensity increased with increase of CNW fillers. The intensity of the peak at 16.4 ° 2θ was decreased for all the composites compared to PLA which suggested a decrease of crystallinity in the composites. The crystallinity of PLA and its composites is summarized in Table 1. Generally, the crystallinity of PLA composites was lower than that of pure PLA (except PLA-CNW2, which was statistically not different from the control, p > 0.05), but even the highest decrease was only about 10%. Although it is shown that the incorporation of a CNW filler impacts the crystallization of PLA, both specific changes in crystal formation and how these changes impact mechanical performance of CNW-PLA composites require further study.

## Conclusions

In this study, amphiphilic whole milk casein was explored as a dispersant in PLA-CNW composites. The SPR study demonstrates the binding of casein to PLA. The binding affinity was 5.40 × 10^4^ M^-1^. The pure PLA and PLA composite samples were prepared using a solvent casting method. The dispersion of CNWs in PLA was studied by Calcofluor staining. The results show the dispersion of CS-CNWs in PLA matrix was better than unmodified CNWs. The evaluation of the mechanical properties of pure PLA and its composites shows the reinforcement effect of CNWs. The Young’s modulus of PLA composites with 10% CS-CNW was 40% higher than that of the pure PLA. CS-CNW composites with 2% filler loading maintained high strain and stress at break. However, the tensile strength and strain at break were decreased with 5% or 10% CS-CNW loading. Most PLA composites exhibited lower crystallinity than pure PLA. Smaller and more numerous spherulites were also observed in PLA-CS-CNW composites demonstrating the improved CNW dispersion. Based on the results of this study, casein may be applied as a dispersant and binder in different composite systems such as those involving clays and minerals, as well as fibers, due to the binding ability of casein to such materials.

## Materials and methods

### Materials

PLA oligomer (OLYGOs Bioresin 120, MW: 2500-3500 Da, NatureWorks™ LLC) was used for SPR studies. High molecular weight PLA (NatureWorks™ 4032D, MW: 195,000-205,000 Da) was used to make the composites. Whole milk casein proteins (from bovine milk, Sigma) were used as CNW dispersants in the composites. Dimethylsulfoxide (DMSO, Burdick & Jackson), dimethylformamide (DMF, Macron Chemical), sulfuric acid (Mallinckrodt Baker) and sodium hydroxide (EMD chemicals Inc) were all A.C.S. grade and used as received. The SPR chips (18 mm × 18 mm × 1 mm with 10 angstroms of Cr followed by 500 angstroms of Au on standard float glass) were from EMF Corporation. Cellulose from cotton (Whatman™ CF11) was used to prepare CNWs. Calcofluor White Stain (Sigma) was used to stain CNWs. It consisted of Calcofluor White M2R (1 g/L) and Evans Blue (0.5 g/L).

### SPR imaging

The SPR chips were soaked in Piranha solution (sulfuric acid: 30% hydrogen peroxide = 3:1, v/v) for 10–15 minutes to remove any surface contamination before use. Caseins were dissolved in the SPR running buffer (PBS: DMSO = 4:1, v/v, pH = 7.3) until saturation (~0.1 mg/mL). 1 mM PLA solution was prepared in buffer and diluted to 0.01, 0.025, 0.05, 0.075, 0.1, 0.2, 0.5 mM to study the binding of PLA to a casein coated SPR chip.

The SPR imaging system (GWC Technologies, SPRimager®) was used to detect the binding between PLA and casein. The SPR buffer was first flowed across the surface followed by the casein solution. Subsequently, the surface was exposed to the SPR buffer, PLA solution, and then the SPR buffer to study the affinity of PLA to casein.

### SPR data analysis

An SPR data analysis software (V++, Digital Optics) was used to collect all SPR images and process the data. The software was able to average the signal intensity from selected regions of interest on the chip. The percent reflectivity values were subsequently obtained as follows (from GWC Technologies Technical Note 125):

(1) $$\Delta \%R=\frac{100\times \text{SPR}\;\text{value}\times 0.85\times \left(1‒d\right)}{s‒\text{pol}\;\text{pixel}\;\text{intensity}}$$

SPR value was obtained under p-polarized light. In order to convert the SPR value to the reflectivity, the intensity for s-polarized light (s-pol pixel intensity) was obtained for regions of interest. d is the density of the filter used when obtaining s-pol pixel intensity (in this system, d = 0.75). A correction factor (0.85) adjusts for the differential reflectivity of p-polarized and s-polarized light as it traverses the prism and reflects off the gold. Adsorption coefficient for the binding of PLA to casein surfaces was determined by Langmuir isotherm:

(2) $$\Gamma =\Gamma \max\frac{\text{Kc}}{1+\text{Kc}}$$

where *K* = Langmuir equilibrium constant or adsorption coefficient, *c* = PLA concentration, Γ = surface coverage of adsorbed molecules and Γ_max_ = maximum surface coverage as c increases. Change of reflectivity value (Δ%R) reflects the change of surface coverage of adsorbed molecules (Γ). The curve fitting was performed with Origin 8.0 (Origin Lab Corporation) using a hyperbola function.

### CNW production

The method by Bondeson et al. [36] was used to prepare CNWs. Generally, CF11 cellulose of 55 g was hydrolyzed in 500 mL 63.5% w/w sulfuric acid at 45°C for 90 min. The reaction was stopped by putting the container on ice until the temperature dropped to 15°C. The excess sulfuric acid was removed by centrifugation (10 min, 16,000 g). The supernatant was removed and replaced by DI water. Centrifugation and washing steps were repeated until the pH of the solution reached 3. The pH of the suspension was then increased to 7 using 0.1 M sodium hydroxide. Finally, the CNW suspension was dialyzed against deionized (DI) water and sonicated to help dispersion of the CNWs (10 min, Branson Model 5510, Danbury). The suspension formed two layers after sedimentation over a few days. The top layer in which CNWs were suspended was used for this study.

### Field emission scanning electron microscopy

CNWs were air dried and sputter coated with a thin gold film (approximately 5 nm) to avoid charging. A LEO 1530 field emission scanning electron microscope (Leo Co., Oberkochen, Germany) was operated at 5 kV to study the morphology of the CNWs.

### Adsorption of casein to CNWs

The amount of adsorption of casein to CNWs was studied using the Bradford method [37]. A standard protein concentration curve was generated by plotting absorbance at 595 nm versus known bovine serum albumin protein solution concentration (UV-2250 UV–VIS spectrophotometer, Shimadzu Corp., Japan). Whole milk casein was dissolved in sodium hydroxide (pH = 10) to make a 1 mg/mL solution. Approximately 50 μg/mL and 100 μg/mL casein solution was obtained by diluting a 1 mg/mL solution. It was shown that the suspension of casein in alkaline solutions can disrupt the micelle [38]. 100 mg freeze dried CNWs were mixed with 4 mL casein solutions overnight with mechanical stirring. Subsequently, the CNWs suspension was centrifuged at 16,000 g for 10 min. The unbound casein was left in the supernatant and the protein concentration of supernatant was determined. The difference of protein concentration before and after mixing to CNWs was converted to the amount of casein adsorbed to CNWs. For 50 μg/mL casein solution, the adsorption of casein to CNWs was 0.92 ± 0.07 μg_casein_/mg_CNW_. For 100 μg/mL casein solution, the adsorption was 1.20 ± 0.13 μg_casein_/mg_CNW_. Casein solution with higher concentration was also used and the amount of adsorption did not increase significantly. The adsorption of casein to CNWs should approach the saturation point at roughly 0.1% w/w casein/CNW.

To make casein functionalized CNWs (CS-CNW), freeze dried CNWs were mixed with casein solution (pH = 10) overnight with mechanical stirring. The concentration of whole milk casein was selected to ensure saturation of the binding sites on CNWs as shown above. The CNWs suspension was centrifuged at 16,000 g for 10 min and the supernatant was replaced with DI water three times to remove any unbound casein.

### Preparation of PLA-CNW composites

Freeze-dried CNWs and CS-CNWs were re-suspended in DMF by mechanical stirring and sonication. The suspensions were stable and no precipitation was observed over three days. High molecular weight PLA was dissolved in the suspension at 100°C forming a 5 wt% solution. Pure PLA samples were also dissolved in DMF. Composites with 2%, 5% and 10% CNW or CS-CNW were casted on a flat glass plate, allowing the DMF to evaporate at 100°C overnight and then dry in a vacuum oven for 24 h at 50°C. The resulting samples were used for all the studies below. Typical film thicknesses were approximately 50 μm. The samples were stored in vacuum oven at room temperature until testing.

### Fluorescence microscopy

The composite samples were characterized using an optical microscope (Zeiss Axio imager A1m). The samples were examined on a clean glass slide. For fluorescence image, a drop of Calcofluor White Stain and a drop of 10% sodium hydroxide were added to the sample and a cover slide was placed over the specimen. The sample was undisturbed for 20 min and then examined under UV light. A 350 nm bandpass optical filter was used to filter only the Calcoflour fluorescence for imaging of the cellulose. The sodium hydroxide was used to slowly dissolve the PLA to expose the cellulose for staining.

### Tensile testing

A dynamic mechanical analyzer (Model Q800, TA Instruments) were used to study the mechanical properties of pure PLA and PLA composites. The typical sample dimension for evaluation of tensile modulus was 25 mm × 5 mm × 50 μm. The actual dimension of the samples was measured by caliper and recorded. The test was performed at a strain ramp speed of 5 mm/min until break at a temperature of 35°C. Tensile strength (MPa), strain at break (%), and the Young’s modulus (GPa), which was calculated from the data of stress and strain in the initial linear region, were recorded. For each material, at least five samples were used to obtain the average value and standard deviation. The significant difference of the data was evaluated using T-tests (with p < 0.05 confident interval).

### X-ray diffraction

X-ray diffraction (XRD) experiments (PANalytical Empyrean) with CuKα radiation generated at 45 kV and 40 mA was used to study the crystallinity of PLA and PLA composites. XRD data were obtained at a rate of 2 degrees per minute from 5 to 50 degrees 2θ. Four major crystalline peaks and one amorphous peak (fixed at 17 degrees 2θ) of PLA were deconvoluted using Peakfit (http://www.systat.com). In the PLA composites, the cellulose peak was assigned at 22.7 degrees 2θ. Gaussian function was used to fit the peaks. The crystallinity of PLA was calculated as the area of PLA crystalline peaks divided by the total area of PLA peaks (the area under the cellulose peak was not included).

## Competing interests

The authors declare that they have no competing interests.

## Authors’ contributions

JG carried out the experiments and data analysis. JC conceived of the study and participated its design. Both authors read and approved the final manuscript.
